# Determination of the Novel Insecticide Flupyradifurone and Its Two Metabolites in Traditional Chinese Herbal Medicines Using Modified QuEChERS and High-Performance Liquid Chromatography-Tandem Mass Spectrometry

**DOI:** 10.1155/2020/8812797

**Published:** 2020-11-12

**Authors:** Nan Fang, Zhou Lu, Zhongbei Zhang, Zhiguang Hou, Shuang Liang, Bo Wang, Zhongbin Lu

**Affiliations:** ^1^College of Plant Protection, Jilin Agricultural University, Changchun, Jilin 130118, China; ^2^Laboratory of Quality & Safety Risk Assessment for Ginseng and Antler Products, Jilin Agricultural University, Changchun, Jilin 130118, China

## Abstract

In this study, an analytical method for the simultaneous determination of the novel insecticide flupyradifurone and its two metabolites in a variety of traditional Chinese herbal medicines was developed for the first time using high-performance liquid chromatography-tandem mass spectrometry. A simple and efficient method using dispersive solid-phase extraction was employed for the pretreatment of the samples. Several extractions and cleanup strategies were evaluated. The recoveries (*n* = 15) of flupyradifurone and its metabolites at three spiking levels were in the range 71.3%–101.7%, with corresponding intraday and interday relative standard deviations of 1.1%–14.8%. The limits of quantitation were 0.01 mg/kg for flupyradifurone and 0.1 mg/kg for its two metabolites. Overall, our developed method was sensitive and reliable for the fast screening of flupyradifurone and its two metabolites in traditional Chinese herbal medicine samples.

## 1. Introduction

Flupyradifurone (IUPAC name, 4-((6-chloro-3-pyridylmethyl) (2,2-difluoroethyl)amino)furan-2(5H)-one, referred to as FPO in this paper) is a novel butenolide insecticide developed by Bayer with systemic action and low mammalian toxicity. FPO is used to control a variety of sucking pests in agricultural and horticultural crops and is extremely effective against pests resistant to neonicotinoid insecticides. FPO is the first nicotinic acetylcholine receptor (nAChR) insecticide containing the stemofoline-derived (natural compound) butenolide pharmacophore [1]. Moreover, it has no metabolic cross-resistance with other nicotinic acetylcholine receptor agonists and is classified as a new subgroup (4*D*) by the International Action Committee on Pesticide Resistance. As recommended by the Joint FAO/WHO Meeting on Pesticide Residues (JMPR) and European Food Safety Authority (EFSA), the residue definition of FPO for estimation of dietary intake for plant commodities is the sum of FPO, difluoroacetic acid (DFA), and 6-chloronicotinic acid (6-CNA), expressed as parent equivalents [2, 3]. Molecular structures and chemical information of FPO and its two metabolites are shown in Table S1.

Traditional Chinese herbal medicines (TCHMs) play a very important role in the Chinese medical system. Many varieties of TCHM are employed, most of which are currently artificially planted. To ensure the quality and yield of TCHM crops, pesticides are widely used in their cultivation to protect them from pests and diseases. However, not only does the excessive or incorrect use of these chemicals cause environmental pollution, but the resulting pesticide residues in TCHMs may also affect the health of consumers. Additionally, with the increasing popularity of TCHMs worldwide, many organizations, including the Food and Agriculture Organization (FAO) and the European Union (EU), have set maximum residue limits (MRLs) for some pesticides in TCHMs and related products.

In studies used to set MRLs, the QuEChERS (Quick, Easy, Cheap, Effective, Rugged, and Safe) method developed by the US Department of Agriculture (USDA) in 2003 is widely used [4, 5]. Because of its flexibility, the QuEChERS method has been successfully used for the analysis of pesticide residues in various matrices [6–13], including TCHMs, in recent years [14–16].

To the best of our knowledge, only Li et al. [17] have reported an analytical method for the detection of FPO, difluoroethyl-amino-furanone (DFEAF), and 6-CNA in fruits, vegetables, and grains. However, both the JMPR and EFSA include DFA in the residue definition for the monitoring of FPO in foods of plant and animal origin [2, 3]. Therefore, the development of an analytical method for DFA is urgently required. In this study, we developed an analytical method for the simultaneous determination of FPO, DFA, and 6-CNA in four representative TCHMs using a modified QuEChERS method and HPLC–MS/MS. The selected TCHMs included a flower (*Lonicerae japonicae* Flos, the dried flower bud of *Lonicera japonica* Thunb.), leaf (*Perillae folium* Perilla, the dried leaves of *Perilla frutescens* (L.) Britt.), root (*Ginseng radix* Et Rhizoma, the dried roots and rhizomes of *Panax ginseng* C. A. Mey.), and whole herb (*Leonuri herba*, the dried aboveground part of *Leonurus japonicus* Houtt.) products. The sample preparation procedures were optimized to achieve satisfactory recoveries, matrix effects, and chromatograms. This method could serve as a fast and sensitive approach for the screening of FPO and its metabolites in TCHMs.

## 2. Materials and Methods

### 2.1. Chemicals and Reagents

Flupyradifurone (purity > 99.5%), difluoroacetic acid (DFA, purity > 98.0%), and 6-chloronicotinic acid (6-CNA, purity > 99.2%) were purchased from Chem Service (West Chester, PA, USA).

HPLC-grade methanol and acetonitrile were purchased from MREDA (Beijing, China). Formic acid (FA, purity > 99.0%) was obtained from Sigma-Aldrich (St. Louis, MO, USA). Ultrapure water was prepared using a Milli-Q system (Bedford, MA, USA). The sorbents primary-secondary amine (PSA), octadecyl silica (C_18_), graphitized carbon black (GCB), and polar enhanced polymer-2 (PEP-2) were purchased from Agela Technologies (Tianjin, China). Sodium chloride (NaCl) and anhydrous magnesium sulfate (MgSO_4_) were obtained from Beijing Chemical Reagent Company (Beijing, China).

Stock solutions containing 1,000 mg/L FPO, 6-CNA, or DFA were prepared in methanol. The stock standards were placed in 10 mL amber bottles and stored in a refrigerator at 4 ± 3°C. Under these storage conditions, the stock standards were replaced after three months.

### 2.2. Sample Preparation

Samples of four TCHMs (*Ginseng radix Et Rhizoma*, *Lonicerae japonicae Flos*, *Leonuri herba*, and *Perillae folium Perilla*; referred to as *a*, *b*, *c*, and *d* in this work) were purchased from a pharmacy in Changchun, China and identified by Wencong Liu (Professor, College of traditional Chinese medicine, Jilin Agricultural University). Each sample (dried) was crushed into a fine powder using an electric grinder (Taisite, Tianjian, China). In brief, 2.0 g of a sample was weighed into a 50 mL Teflon centrifuge tube, and 0.1 mL of a standard working solution was added. After 1 min, 10 mL of acetonitrile containing 2% formic acid was added. The tube was then vortexed for 2 min and subjected to centrifugation at 4751.5 g for 5 min. Subsequently, 1.0 mL of the supernatant was transferred to a 4 mL centrifuge tube containing the sorbent and 150 mg of anhydrous MgSO_4_ (*a* was purified using 50 mg of C_18_; *b* and *c* were purified using 50 mg of C_18_ and 10 mg of PSA; *d* was purified using 50 mg of C_18_ and 50 mg of GCB), and vortexed for 30 s before being centrifuged at 4751.5 g for 5 min. Afterward, 0.5 mL of the supernatant was transferred to a 2 mL centrifuge tube and dried using a gentle stream of nitrogen. Finally, 0.5 mL of a mixture consisting of acetonitrile and water (acetonitrile:water = 1 : 4, v/v) was added to reconstruct the sample. The final solution was filtered through a 0.22 *μ*m nylon syringe filter into a 2 mL vial for analysis by HPLC–MS/MS.

### 2.3. Instrumental

An Agilent 1260 HPLC (Santa Clara, CA, USA) equipped with an Agilent ZORBAX RRHD Eclipse Plus C_18_ column (3.0 × 100 mm, 1.8 *μ*m) was used for the chromatographic separation. The column oven was maintained at 30°C. The mobile phase consisted of water acidified with 0.1% formic acid (phase A) and acetonitrile (phase B). An isocratic flow (20% B) was employed at a flow rate of 0.3 mL/min. The injection volume was 5 *μ*L. The overall runtime of the analysis was 10.0 min.

The HPLC system was coupled to an Agilent 6470 triple quadrupole mass spectrometer (Santa Clara, CA, USA) equipped with an electrospray ion source (ESI). The ionization of FPO and its metabolites was performed in the positive and negative ionization modes. The following source parameters were used: Nebulizer pressure: 30 psi; capillary and nozzle voltage for positive and negative modes: 4.0 kV and 0.5 kV, respectively; desolvation (drying gas) and sheath gas flow rates: 7 and 8 L/min, respectively; desolvation and source temperatures: 330 and 300°C, respectively.

### 2.4. Method Validation

Based on the SANTE/11813/2017 guidelines [18], the developed method was validated in terms of recovery (accuracy) and intraday and interday repeatability (precision) by spiking blank samples at three levels (0.01, 0.05, and 0.5 mg/kg for FPO; 0.1, 0.5, and 5 mg/kg for DFA and 6-CNA), and each spiking level was replicated five times. The limit of quantitation (LOQ) is defined as the lowest spiking level of the analyte in the matrix.

The quantitation of FPO was performed using the external standard method. Because of the influence of matrix effects (ME) on quantitation during LC-MS analysis [19], a seven-point (0.001, 0.005, 0.01, 0.05, 0.1, 0.5, and 1 mg/L) matrix-matched calibration curve was used instead of a curve comprised of solvent standards for quantitation to account for any signal enhancement or suppression caused by ME. The extent of the ME on the analyte ion abundance in different matrices was measured using the following equation:

(1) $$\begin{array}{c} \text{ME}\%=\left(\frac{\text{slope of matrix}-\text{matched calibration curve}}{\text{slope of solvent calibration curve}}-1\right)\times 100\%. \end{array}$$

## 3. Results and Discussion

### 3.1. Optimization of MS/MS Parameters

The MS/MS parameters of the three compounds were investigated in both ESI^+^ and ESI^−^ modes. The results showed that DFA and 6-CNA had higher responses in the negative mode, while FPO showed a higher response in the positive mode.

The MS/MS parameters that could affect the intensity of the ion transitions, including the fragmentor voltage and collision energy (CE), were optimized by ramping the values of each parameter over a certain range and selecting the value that yielded the highest signal response. The selected ion pairs and optimized MS/MS parameters are listed in Table S2.

### 3.2. Optimization of Chromatography

**Figure 1:**
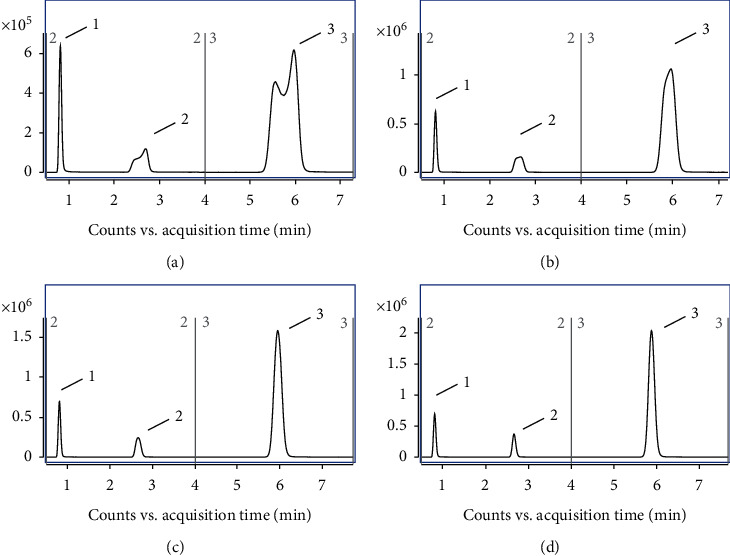
HPLC-MS/MS chromatograms of DFA (1.0 mg/L, peak 1), 6-CNA (1.0 mg/L, peak 2), and flupyradifurone (0.1 mg/L, peak 3) on C_18_ column and the same elution procedure under different injection solutions conditions. ((a) Acetonitrile: water = 1 : 0 (v/v), (b) acetonitrile: water = 4 : 1 (v/v), (c) acetonitrile: water = 1 : 1 (v/v), (d) acetonitrile: water = 1 : 4 (v/v)).

The mobile phase for the chromatographic separation was optimized to achieve sufficient peak separation with high sensitivity and for the switch between the negative and positive ESI modes. Gradient elution on the C_18_ column with methanol-water and acetonitrile-water as the mobile phase was used to separate the three target compounds. The retention time of FPO was shorter when the proportion of methanol or acetonitrile in the mobile phase was increased, and the retention of FPO was affected when the proportion of methanol or acetonitrile in the mobile phase was less than 20%. The proportions of phases A and B had little effect on 6-CNA and almost no effect on DFA. The separation of the three compounds met the requirements for positive and negative mode switching when the mobile phase was acetonitrile : water = 1 : 4 (v/v). However, the solvent effect affected the peak shapes of DFA and 6-CNA. Therefore, the addition of different proportions of water to the injection solutions to eliminate the solvent effect was evaluated. The results (Figure 1) show that the peak shape was improved when the ratio of acetonitrile to water in the injection solutions was close to that of the mobile phase. Additionally, Stahnke et al. [20] reported that matrix effects can be reduced by dilution of the sample extracts and that the hydrophobic organic coextracts in the sample extract are diluted when injection solutions with a high proportion of water are used. This strategy was helpful in reducing the matrix effect. Therefore, the injection solution composition was set as acetonitrile : water = 1 : 4 (v/v).

**Figure 2:**
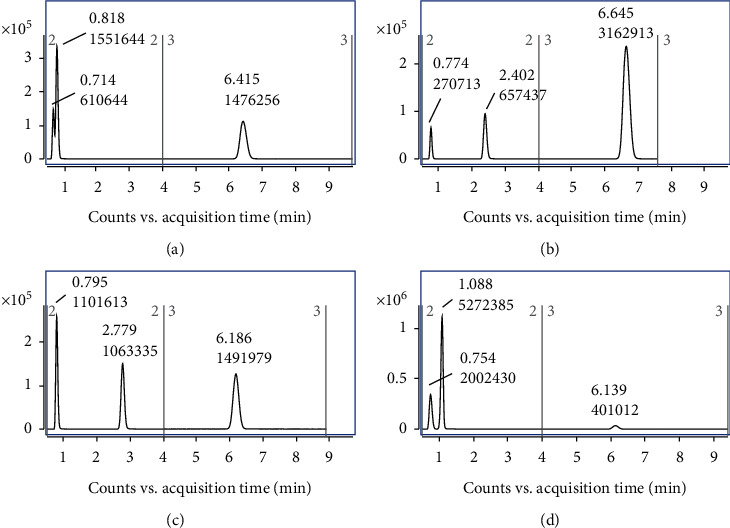
HPLC-MS/MS chromatograms of DFA (1.0 mg/L), 6-CNA (1.0 mg/L) and flupyradifurone (0.1 mg/L) on C_18_ column and the same elution procedure under different mobile phase conditions. ((a) 5 mmol ammonium acetate aqueous solution/acetonitrile, (b) 0.1% formic acid and 5 mmol ammonium acetate aqueous solution/acetonitrile, (c) 0.1% formic acid aqueous solution/acetonitrile, (d) water/acetonitrile).

In addition, the effect of the addition of 0.1% formic acid, 5 mmol ammonium acetate, or both on the sensitivity of the three compounds was studied. The results (Figure 2) show that the ionization efficiency of FPO was enhanced by the addition of formic acid and ammonium acetate, while the ionization efficiencies of DFA and 6-CNA were inhibited by the addition of either. Through analysis of the peak areas of the three compounds, we found that the sensitivity of the three compounds was acceptable when 0.1% formic acid was added to the mobile phase.

### 3.3. Optimization of Extraction

**Figure 3:**
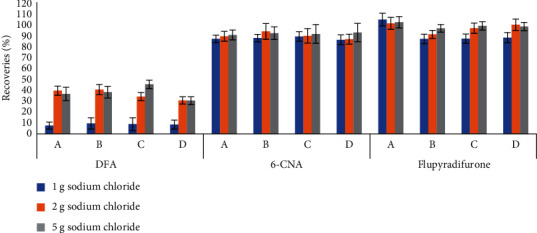
Recovery of flupyradifurone, DFA, and 6-CNA for the improved method under different amounts of sodium chloride condition (*n* = 3).

**Figure 4:**
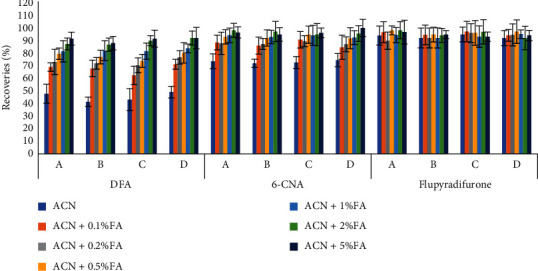
Recovery of flupyradifurone, DFA, and 6-CNA for the method using different concentrations of formic acid (*n* = 3).

The improved QuEChERS method (with the addition of 10 mL of water) has been recommended for the determination of pesticide residues in dried samples. However, the recoveries of DFA using this method were less than 10%, and no significant change was observed after adjusting the pH. This indicated that the partitioning of DFA into acetonitrile was difficult because of its high solubility in water. To solve this problem, different amounts (1, 2, or 5 g) of NaCl were added to the extraction solution. The efficiency of the solvent extraction process can be improved by salt [21], as the addition of salt reduces the solubility of the analyte in an aqueous solution and strengthens the partitioning of the analyte into the organic phase. On the other hand, salt increases the viscosity of the aqueous phase and reduces the diffusion coefficient of analyte in the aqueous phase and the solubility of the extraction solvent in the aqueous phase. The effects of the addition of different amounts of NaCl on the recoveries are shown in Figure 3. NaCl addition did enhance the extraction efficiency of DFA, but the recoveries of DFA were still less than 70% even when 5 mL of ultrapure water saturated with NaCl was used. This indicated that the addition of NaCl could not achieve the complete partitioning of DFA into acetonitrile. Therefore, the samples were extracted using only acetonitrile with different concentrations of formic acid (0.1%–5.0%) in the final extraction method test. The results (Figure 4) showed that the recoveries of DFA and 6-CNA increased significantly with the formic acid concentration in the extract up to a formic acid concentration of 2%. Therefore, acetonitrile containing 2% formic acid was chosen as the extractant. This modified QuEChERS extraction method is simple and fast, and the water-free extraction process reduces the coextraction of hydrophilic organic compounds.

### 3.4. Optimization of Purification

The choice of a suitable purification step is very important to obtain accurate results using LC–MS/MS and GC–MS/MS. Matrix extracts often contain many polar organic coextracts (including organic acids and aliphatic acid) and nonpolar organic coextracts (such as fats and pigments). These organic coextracts will enhance the matrix effect, increase the background in the mass spectrum, and pollute the ion source [22]. The purpose of purification is to reduce the matrix effect and background as much as possible. However, the target compounds are also inevitably adsorbed when sorbents are used to remove the polar or nonpolar organic coextracts. Therefore, controlling the balance between recovery and the removal of undesirable matrix components in the purification process is important.

**Figure 5:**
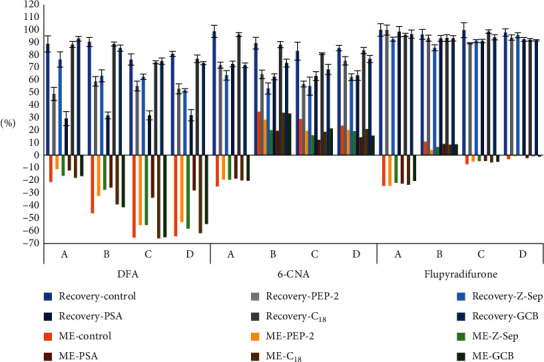
Recovery and ME of flupyradifurone, DFA, and 6-CNA for the method using different sorbents (*n* = 3).

In this paper, the purification effects of several common sorbents on DFA, 6-CNA, and FPO in dried samples of *Ginseng radix Et Rhizoma*, *Lonicerae japonicae Flos*, *Leonuri herba*, and *Perillae folium Perilla* (referred to as *a*, *b*, *c*, and *d*, respectively) were evaluated. The sorbents PSA, C_18_, PEP-2, Z-Sep, and GCB were selected, and 50 mg of each sorbent and 150 mg of anhydrous MgSO_4_ were used for the purification of 1 mL of the extract solution. The effect of these sorbents on the recovery is shown in Figure 5. DFA was adsorbed on all the sorbents except for C_18_ and GCB, with its adsorption on PSA being strongest. Chermahini et al. [23] found that DFA has only three stable conformations; *cis* DFA has two stable conformers, EHC and EFC, while *trans* DFA has one stable conformer, EHT. The stability of the DFA monomers follows the order EHC > EFC > EHT. Additionally, a comparison of the hydrogen bonding strength of DFA with those of acetic acid and fluoroacetic acid shows that the replacement of one of the electron-withdrawing groups with a hydrogen atom results in a decrease in its hydrogen bonding strength. PSA has a strong chelating effect due to the primary and secondary amino groups in its structure [24]. Therefore, a likely reason for the decrease in the DFA recovery was the formation of a chelate between DFA and PSA. This phenomenon was consistent with the results of Li et al. [17]. The purification effects of all the sorbents were evaluated via the matrix effect. The results (Figure 5) showed that the matrix effects of PSA on DFA and 6-CNA were significantly lower than those of the other sorbents, because DFA and 6-CNA are polar compounds, and PSA retains free fatty acids and other polar disrupters in the matrix. The use of GCB significantly reduced the matrix effects for *b*, *c*, and *d*, which contained more pigments.

**Figure 6:**
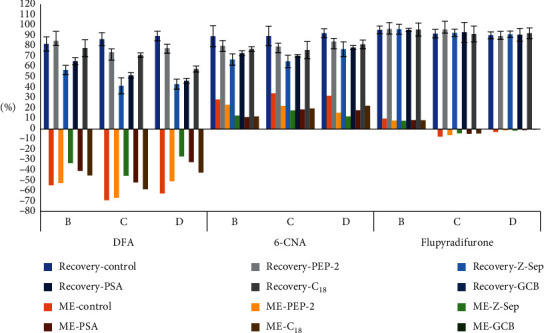
Recovery and ME of flupyradifurone, DFA, and 6-CNA for the method under different combinations of sorbent conditions (*n* = 3).

The recoveries of the three compounds in all the matrices only reached greater than 70% when C_18_ was used. However, the matrix effects for *b*, *c*, and *d* purified using only C_18_ were still unsatisfactory. Li et al. [17] also reported that the recoveries of 6-CNA and FPO were satisfactory after purification using C_18_, but the matrix effects were not. This was likely because C_18_ cannot remove polar organic coextracts and pigments very well. Therefore, although purification with PSA reduced the recoveries of DFA and 6-CNA, its good purification effect made it the best choice. To further reduce the matrix effects for *b*, *c*, and *d*, GCB and PSA were used in combination with C_18_ for purification. The following sorbent combinations were evaluated: 50 mg C_18_ + 50 mg GCB (combination 1), 50 mg C_18_ + 30 mg PSA (combination 2), 50 mg C_18_ + 20 mg PSA (combination 3), and 50 mg C_18_ + 10 mg PSA (combination 4). Each combination contained 150 mg anhydrous MgSO_4_. The purification results are shown in Figure 6. None of the sorbent combinations affected the recovery or matrix effects for FPO in the three matrices. The recoveries of DFA and 6-CNA purified using combination 1 and combination 4 were within the acceptable parameters established by the SANTE guidelines, but the matrix effects were lower for the DFA and 6-CNA purified using combination 4. Therefore, combination 4 was selected to clean up *b* and *c*. For *d*, recovery of more than 70% was only achieved using combination 1. Therefore, combination 1 was selected to clean up *d*.

### 3.5. Method Validation Results

The calibration data, ME, and LOQs of FPO, DFA, and 6-CNA in the different matrices are listed in Table 1. Good linearity (*r* > 0.99) was obtained for all the matrix-matched calibration curves in the range of 0.001–1 mg/L. The LOQs were defined as 0.01 mg/kg for FPO and 0.1 mg/kg for DFA and 6-CNA in all matrices according to the SANTE guidelines.

The mean recoveries (*n* = 15) of FPO, DFA, and 6-CNA from TCHM samples spiked at three levels were in the range 71.3%–101.7%, with intraday and interday relative standard deviations (RSDs) ranging from 1.1% to 14.8% and from 1.2% to 5.5%, respectively. More detailed data are provided in Table S3.

### 3.6. Application of the Method in Real Samples

To confirm the applicability of the developed and validated method, the method was applied for the determination of FPO, DFA, and 6-CNA in 10 commercial TCHMs (*Ginseng radix Et Rhizoma*, *Astragali radix*, *Isatidis radix*, *Lonicerae japonicae Flos*, *Artemisiae Argyi Folium*, *Leonuri herba*, *Taraxaci herba*, *Perillae folium Perilla*, *Schisandrae chinensis Fructus*, and *Siraitiae fructus*, *n* = 5 for each) from Jilin Province, China. FPO, DFA, and 6-CNA were not found in any of the samples.

## 4. Conclusions

A modified version of the original QuEChERS method was established for the determination of FPO and its two metabolites (DFA and 6-CNA) in dried samples of TCHMs. The three target compounds were successfully separated on a C_18_ column. The samples were extracted using acetonitrile containing formic acid and cleaned up using different combinations of sorbents. This method reduced the extraction of hydrophilic organic coextracts in the extraction process and caused the hydrophobic organic coextracts to precipitate when the extraction solution was diluted to prepare the injection solutions, which reduced the interference with the target compounds. In the validation of the method, satisfactory linearity, repeatability, intermediate precision, and accuracy were obtained. The recoveries of the method were in the 70 to 120% range. The method precision in terms of repeatability and intermediate precision was adequate, with RSD values of <20%. These results demonstrated that the developed method is rapid and reliable for monitoring FPO and its two metabolites (DFA and 6-CNA) in TCHMs.

## Figures and Tables

**Table 1:** Calibration information of target compounds in the range of 0.01–1 mg/L in different matrices.

Compound	Matrix	Calibration equation	*R* ^2^	ME (%)	LOQ (*μ*g/kg)
DFA	Acetonitrile	*y* = 1546357*x* − 4449	0.9999	—	—
	*a*	*y* = 1269154*x* − 2008	0.9998	−17.9	100
	*b*	*y* = 117969*x* − 137	1	−45.0	100
	*c*	*y* = 461557*x* − 1722	0.9997	−58.4	100
	*d*	*y* = 763900*x* − 1223	0.9998	−50.6	100

6-CNA	Acetonitrile	*y* = 1897410*x* − 5191	0.9998	—	—
	*a*	*y* = 1518248*x* − 2979	0.9998	−20.0	100
	*b*	*y* = 174070*x* + 479	0.9999	12.3	100
	*c*	*y* = 1257239*x* − 3696	0.9998	20.0	100
	*d*	*y* = 2197201*x* − 2904	0.9999	15.8	100

FPO	Acetonitrile	*y* = 16016804*x* − 37345	0.9999	—	—
	*a*	*y* = 12279227*x* − 22330	0.9998	−23.3	10
	*b*	*y* = 1447617*x* − 3859	0.9998	8.5	10
	*c*	*y* = 11072572*x* − 34209	0.9998	−4.2	10
	*d*	*y* = 15840619*x* − 27422	0.9999	−1.1	10

## Data Availability

The data used to support the findings of this study are available from the corresponding author upon request.

## Supplementary Materials

Supplementary Materials Table S1: chemical information of flupyradifurone and its metabolites. Table S2: optimized MRM conditions for analysis of flupyradifurone, DFA, and 6-CNA. Table S3: mean recoveries and RSD for target compounds from different matrices at three spiked levels.
