# Supplementary material for: Determination of the Novel Insecticide Flupyradifurone and Its Two Metabolites in Traditional Chinese Herbal Medicines Using Modified QuEChERS and High-Performance Liquid Chromatography-Tandem Mass Spectrometry
Source: Int J Anal Chem. 2020 Nov 12;2020:8812797. doi: 10.1155/2020/8812797 (PMC7895605; doi:10.1155/2020/8812797)
Supplement: Supplementary Materials — Table S1: chemical information of flupyradifurone and its metabolites. Table S2: optimized MRM conditions for analysis of flupyradifurone, DFA, and 6-CNA. Table S3: mean recoveries and RSD for target compounds from different matrices at three spiked levels. [file 8812797.f1.docx]

**SUPPORTING MATERIALS**

Table S1: Chemical information of flupyradifurone and its metabolites.

| Chemical name | Code | Chemical formula | CAS RN | Structural formula |
| --- | --- | --- | --- | --- |
| flupyradifurone | - | C_12_H_11_C_l_F_2_N_2_O_2_ | 951659-40-8 |  |
| difluoroacetic acid | DFA | F_2_C_2_H_2_O_2_ | 381-73-7 |  |
| 6-chloronicotinic acid | 6-CNA | C_6_H_4_C_l_NO_2_ | 5326-23-8 |  |

Table S2: Optimized MRM conditions for analysis of flupyradifurone, DFA and 6-CNA.

| Compound | Ion source | Retention time | Precursor ion | Quantification ion (a) | Confirmation ion (a) | Fragmentor |
| --- | --- | --- | --- | --- | --- | --- |
| Flupyradifurone | ESI+ | 5.84 | 289.0 | 245.1 (15) | 126.1 (25) | 140 |
| DFA | ESI- | 0.82 | 95.2 | 51.1 (10) | 51.1 (10) | 70 |
| 6-CNA | ESI- | 2.64 | 156.0 | 111.9 (8) | 35.1 (10) | 40 |

^a^ collision energy (eV), it is given in parentheses.

Table S3: Mean recoveries and RSD for target compounds from different matrices at three spiked levels.

| Compound | Intraday (n=5)  %Recovery (RSD_r_^a^) | | | | | | | | | | |  | Inter-day (n=15)  %Recovery (RSD_R_^b^) | | | | |
| --- | --- | --- | --- | --- | --- | --- | --- | --- | --- | --- | --- | --- | --- | --- | --- | --- | --- |
|  | DFA | | |  | 6-CNA | | |  | Flupyradifurone | | |  | DFA |  | 6-CNA |  | Flupyradifurone |
| Spiked level (μg/kg) | 100 | 500 | 5000 | | 100 | 500 | 5000 | | 10 | 50 | 500 | | 500 | | 500 | | 50 |
| a | 90.3 (2.6) | 85.7 (1.5) | 84.0 (0.8) | | 93.7 (3.3) | 97.3 (1.4) | 93.3 (2.2) | | 95.7 (2.7) | 94.1 (1.5) | 92.6 (1.5) | | 90.2 (1.8) | | 97.3 (2.1) | | 95.9 (1.7) |
| b | 84.7 (3.4) | 75.8 (2.0) | 78.2 (0.8) | | 81.6 (1.7) | 78.0 (1.1) | 75.4 (1.9) | | 100.3 (4.1) | 92.0 (1.9) | 92.7 (2.3) | | 78.7 (2.0) | | 79.4 (1.9) | | 93.2 (1.7) |
| c | 82.9 (5.2) | 71.3 (1.7) | 73.3 (1.1) | | 83.2 (1.8) | 78.5 (1.7) | 79.2 (1.3) | | 94.7 (2.5) | 94.0 (2.3) | 96.3 (1.5) | | 72.9 (2.3) | | 81.8 (2.2) | | 93.7 (2.1) |
| d | 82.9 (4.4) | 73.8 (2.2) | 73.8 (1.2) | | 94.5 (11.2) | 85.2 (14.8) | 88.1 (8.0) | | 97.6 (1.7) | 96.2 (1.6) | 94.6 (1.3) | | 75.2 (2.6) | | 87.5 (5.5) | | 97.2 (2.5) |

^a^ the intraday relative standard deviation for repeatability (n=5)

^b^ the inter-day relative standard deviation for repeatability (n=15)
